# Which environmental factors most strongly influence a street’s appeal for bicycle transport among adults? A conjoint study using manipulated photographs

**DOI:** 10.1186/s12942-016-0058-4

**Published:** 2016-09-01

**Authors:** Lieze Mertens, Delfien Van Dyck, Ariane Ghekiere, Ilse De Bourdeaudhuij, Benedicte Deforche, Nico Van de Weghe, Jelle Van Cauwenberg

**Affiliations:** 1 Department of Movement and Sport Sciences, Faculty of Medicine and Health Sciences, Ghent University, Watersportlaan 2, 9000 Ghent, Belgium; 2 Research Foundation Flanders (FWO), Egmontstraat 5, 1000 Brussels, Belgium; 3 Department of Public Health, Faculty of Medicine and Health Sciences, Ghent University, De Pintelaan 185, 4k3, 9000 Ghent, Belgium; 4 Department of Human Biometry and Biomechanics, Faculty of Physical Education and Physical Therapy, Vrije Universiteit Brussel, Pleinlaan 2, 1050 Brussels, Belgium; 5 Department of Geography, Faculty of Sciences, Ghent University, Krijgslaan 281, S8, 9000 Ghent, Belgium

**Keywords:** Active transport, Micro-environment, Built environment, Biking, Adulthood, Experiment, Photographs

## Abstract

**Background:**

Micro-environmental factors (specific features within a streetscape), instead of macro-environmental factors (urban planning features), are more feasible to modify in existing neighborhoods and thus more practical to target for environmental interventions. Because it is often not possible to change the whole micro-environment at once, the current study aims to determine which micro-environmental factors should get the priority to target in physical environmental interventions increasing bicycle transport. Additionally, interaction effects among micro-environmental factors on the street’s appeal for bicycle transport will be determined.

**Methods:**

In total, 1950 middle-aged adults completed a web-based questionnaire consisting of a set of 12 randomly assigned choice tasks with manipulated photographs. Seven micro-environmental factors (type of cycle path, speed limit, speed bump, vegetation, evenness of the cycle path surface, general upkeep and traffic density) were manipulated in each photograph. Conjoint analysis was used to analyze the data.

**Results:**

Providing streets with a cycle path separated from motorized traffic seems to be the best strategy to increase the street’s appeal for adults’ bicycle transport. If this adjustment is not practically feasible, micro-environmental factors related to safety (i.e. speed limit, traffic density) may be more effective in promoting bicycle transport than micro-environmental factors related to comfort (i.e. evenness of the cycle path surface) or aesthetic (i.e. vegetation, general upkeep). On the other hand, when a more separated cycle path is already provided, micro-environmental factors related to comfort or aesthetic appeared to become more prominent.

**Conclusions:**

Findings obtained from this research could provide advice to physical environmental interventions about which environmental factors should get priority to modify in different environmental situations.

**Trial registration:**

The study was approved by the Ethics Committee of the Ghent University Hospital. Trial registration: B670201318588. Registered at 04/10/2013. http://www.ugent.be/ge/nl/faculteit/raden/ec

**Electronic supplementary material:**

The online version of this article (doi:10.1186/s12942-016-0058-4) contains supplementary material, which is available to authorized users.

## Background

Although cycling is known as a sustainable form of human transport, it is not yet sufficiently integrated into daily life routines in the global population. In Europe, 50 % of all trips are shorter than 3 km, which is a feasible distance for cycling. However, a large part of these trips is still done by motorized modes of transport [1]. For example in Flanders (Belgium), only 25 % of all trips shorter than 3 km and only 14 % of all trips shorter than 5 km are done actively (i.e. by foot or by bike) among adults between 18 and 65 years old [2]. Several cross-sectional studies among adults indicated that bicycle transport is associated with higher general physical activity levels and lower body weight [3–6]. In addition, bicycle transport also has many other benefits on social (social cohesion), environmental (reduced carbon footprint) and economic (infrastructure costs) level [7–14]. It is therefore in favor of both the individual and the community to create supportive environments that make it easier to engage in bicycle transport [15–18]. Policy development together with relevant sectors such as urban planning, active transport policies, built environment strategies and crime prevention polices should be encouraged at national and subnational level to promote regular bicycle transport by adapting the environment or community [19–24]. By modifying the environment, large populations over long periods of time can be reached. It is therefore important to know which environmental determinants affect bicycle transport among adults.

Built environmental variables can be classified into two broad categories: macro- and micro-scale environmental factors [25, 26]. Macro-environmental factors can be regarded as ‘raw’ urban planning features; such as walkability, connectivity of the street network, residential density and land use mix diversity. These factors are difficult to change in existing environments because of their large size and complexity, and because they are influenced by different levels of authorities [25, 26]. On the other hand, micro-environmental factors can be defined as relatively small environmental factors such as evenness of the cycle path surface, vegetation and speed limits. These factors are influenced by individuals or local actors and are less complex which makes them more feasible to modify in existing neighborhoods (i.e. lower cost and shorter time-frame) compared to the reconfiguration of the macro-scale structural design [25, 26].

In the literature, most research has been conducted on macro-scale environmental factors. Worldwide, consistent strong positive relationships have been found between macro-scale environmental factors and transport-related cycling in adults. Higher levels of walkability, improved access to shops/services/work and higher degree of urbanization were positively related to bicycle transport in adults [27–30]. Unfortunately, research on the micro-environmental factors affecting bicycle transport is scarce and results are inconsistent [31–35]. Previous studies showed inconsistent associations between modifiable micro-environmental factors and bicycle transport [35–38]. For example, some studies found associations of lower road motorized traffic volumes [31] and the presence of traffic calming elements with more cycling for transport [39], while other studies found that higher volumes of motorized traffic were associated with more bicycle transport [36, 38], or found no associations at all [37, 40, 41]. Mixed evidence was also found for aesthetics. Several studies found a positive association between vegetation and bicycle transport [29, 42–44], while other studies did not find significant associations [5, 40, 45]. Furthermore, although the importance of well separated cycle paths for bicycle transport have already been identified [21, 46], not all research could confirm this positive association [37, 47]. Furthermore, it is still unclear which micro-environmental factors relate most strongly to cycling for transport. Because it is often not possible to change the whole micro-environment at once, it is necessary to explore the individual impact of each parameter and to know which environmental factors should get priority in environmental interventions increasing bicycle transport. Furthermore, since the real environment consists of a combination of several environmental factors simultaneously, it is also crucial to investigate the interaction effects of different micro-environmental factors. For example, a previous pilot study (conducted in a small sample) [48] with manipulated photographs showed that the positive effect of cycle path evenness appeared to increase in an environment with good compared to poorly overall upkeep. Conversely, the street’s appeal for bicycle transport decreased when both separations along the cycle path were present (i.e. separation from motorized traffic as well as pedestrians) compared to only a separation with traffic [48]. Furthermore, investigating the relative importance of environmental factors within a particular micro-environmental factor could be interesting for a detailed analysis of these interactions effects. For example, it would be interesting to find out which environmental factors subsequently are important in situations where an even cycle path surface is provided. Unfortunately, this has not frequently been studied in large populations. Therefore, future studies investigating the effect of micro-environmental factors and their interaction effects on the street’s appeal for bicycle transport are important.

The main issue with previous studies investigating the effect of micro-environmental factors on bicycle transport is related to the cross-sectional observational study designs [34, 49]. Although usually valid and reliable tools are used (e.g. questionnaires), there are some methodological concerns: participants have to recall features of the physical environment, which involves recall bias [50] and the lack of standardization in neighborhood definitions increases the inconsistency as well [51]. To accommodate these shortcomings, stronger designs are required with improved causal inference [17, 30, 34, 52, 53]. Since natural experiments are complex, time- and cost-consuming to conduct in real environments, an innovative experimental and cost-effective methodology is required.

Therefore, the present study opts for a controlled experiment: it uses experimental manipulations of environmental factors in photographs to examine whether these factors affect the street’s appeal for bicycle transport. The validity of color photos in comparison to on-site responses has already been proven in previous studies [54, 55]. Furthermore, respondents who judge photographs do not have to recall features of the physical environment (as is the case when using questionnaires), which improves the reliability of the results. In addition, defining the ‘neighborhood’ is no longer necessary with this methodology because the assessment of the physical environment happens consistently between participants. Since these photograph experiments control for co-variation (i.e. environmental factors that co-occur), this approach overpowers previous studies by allowing the researcher to differentiate the separate influence of each environmental factor under controlled conditions [55]. This methodology using manipulated photographs results from previous research with non-manipulated photographs [35] and was tested in a recent mixed-method pilot study investigating the effect of a limited number of key micro-environmental factors and the street’s appeal for adults’ bicycle transport [48]. In this study only five micro-environmental factors were simultaneously manipulated and each factor only had a maximum of two levels. This exploratory study, conducted in a small sample, provided a proof-of-concept to use manipulated photographs to assess a street’s appeal for adults in a controlled experiment. From this previous research step, there is a need to carry out a large-scale study in which the effects of all relevant micro-environmental factors are studied. Findings obtained from these controlled experiments might provide guidelines for interventions that use micro-environmental modifications to create more supportive environments for bicycle transport. Only adults in the age range between 45 and 65 years old where included in this study because they assess the physical environment according to their own needs, rather than in perspective of their parental vision (considering their child).

In summary, this study adds to the literature as it is still unclear what type of infrastructure regarding the micro-environment is required to specifically encourage bicycle transport. Furthermore, the experimental design of our study overpowers previously used cross-sectional observational study designs and moreover is a cost-effective methodology compared to natural experiments. Additionally, one of the main novelties compared to existing literature is that the current study creates an order of importance or hierarchy of the different micro-environmental factors and also investigates interaction effects between different micro-environmental factors.

The main aim of the current study was to determine the relative importance of micro-environmental factors for a street’s appeal for bicycle transport among middle-aged adults (45–65 years). Second, interaction effects among micro-environmental factors on the street’s appeal for bicycle transport were determined to investigate the effect of combinations of micro-environmental factors.

## Methods

### Protocol and measures

By purposeful convenience sampling, Flemish middle-aged adults between 45 and 65 years were recruited using email, social media, family, friends, clubs, organizations and companies. Additional participants were recruited by snowball sampling. Participants completed a two-part web-based questionnaire, which was developed using Sawtooth Software (SSI Web version 8.3.8.). The online questionnaire was available from the beginning of November 2014 until the end of January 2015 and 1969 middle-aged adults completed the study. Eighteen participants who did not have the proper age (45–65 years old) were excluded from the analysis. Informed consent was automatically obtained from the participants when they voluntarily completed the questionnaire. The study was approved by the Ethics Committee of the Ghent University Hospital.

### Photograph development

**Fig. 1:**
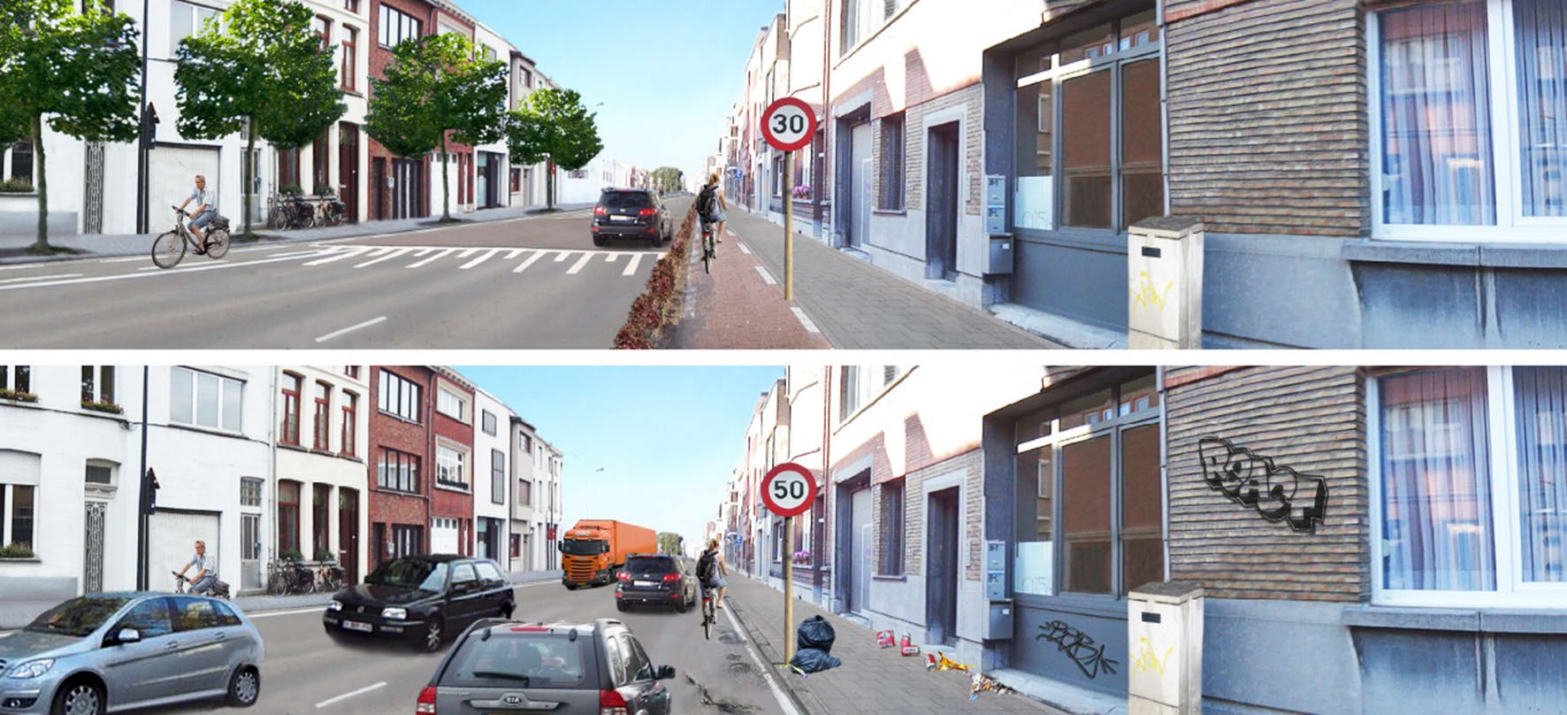
The anticipated best and worst street to cycle along by manipulating the micro-environmental factors (Table 1). Anticipated best street to cycle along (first photograph): C6, S2, B2, V3, E3, M3, D3. Anticipated worst street to cycle along (second photograph): C1, S1, B1, V1, E1, M1, D1

Prior to data collection, a set of 1945 manipulated panoramic color photographs were developed with Adobe Photoshop© software [56]. The developed photographs were all modified versions of one ‘basic’ panoramic photograph representing a typical semi-urban (300–600 inhabitants/km^2^) street in Flanders (Belgium) [57]. The ‘basic’ photograph was taken from an adult cyclist’s eye-level viewpoint under dry weather conditions and depicts a hypothetical cycling route where adults could cycle along. The newly developed photographs differed from each other in at least one micro-environmental manipulation. Seven micro-environmental factors (type of cycle path, speed limit, speed bump, vegetation, evenness of the cycle path surface, general upkeep and traffic density) were manipulated in each photograph and consisted of at least two possible levels. The levels of the environmental factors are presented in Table 1 and the corresponding abbreviations are used throughout the article. These micro-environmental factors and their levels were selected based on existing literature [27, 58] and previous qualitative and quantitative research with (non-)manipulated panoramic photographs [35, 48, 59] studying relationships between the environment and bicycle transport. For example, a previous mixed-methods pilot study with manipulated photographs indicated that it is not inviting for bicycle transport to separate the cycle path and the sidewalk by using bollards [48]. Qualitative data from that study reported that cyclists see these bollards as a disturbing factor that limited their evasive options and also showed that some were afraid to cycle against those bollards. However, from previous research from the Netherlands, Denmark and Germany, we know that it is important to provide a visual and/or physical separation between cyclists and pedestrians for example by grade separation, pavement coloring or surfacing [58]. From this reasoning, we wanted to investigate if a separation by pavement coloration has a more positive effect to separate cyclists from pedestrians instead of bollards as separation. To determine each micro-environmental factor and their levels, a thoughtful reasoning using the literature and previous results was made [27, 35, 48, 58, 59]. An example of the anticipated best and worst street to cycle along are shown in Fig. 1.

**Table 1:** Overview of the manipulated micro-environmental factors and their specific levels

Type of cycle path	C1. No cycle path
	C2. Cycle path, separated from traffic by marked white lines
	C3. Cycle path, separated from traffic with a curb, not separated from walking path by color
	C4. Cycle path separated from traffic with a hedge, not separated from walking path by color
	C5. Cycle path separated from traffic with a curb, separated from walking path by color
	C6. Cycle path separated from traffic with a hedge, separated from walking path by color
Speed limit	S1. 50 km/h
	S2. 30 km/h
Speed bump	B1. Absent
	B2. Present
Vegetation	V1. No trees
	V2. Two trees
	V3. Four trees
Evenness of the cycle path surface	E1. Very uneven surface
	E2. Moderately uneven surface
	E3. Even surface
General upkeep	M1. Bad upkeep (much graffiti and litter)
	M2. Moderate upkeep (a bit of graffiti and litter)
	M3. Good upkeep (no graffiti or litter)
Traffic density	D1. Four cars + truck
	D2. Three cars
	D3. One car

### The web-based questionnaire

The web-based questionnaire consisted of two parts. First, socio-demographic characteristics were assessed: age, gender, country of birth, marital status, education, and occupational status (see Table 2 for the response categories). Self-reported weight and height were assessed to calculate body mass index (BMI). Additionally, the amount of usual bicycle transport in a week was assessed by using the long form of the International Physical Activity Questionnaire (IPAQ: ‘usual week’) [60].

**Table 2:** Descriptive characteristics of the participants (n = 1950)

Age (M ± SD) (years)	54.3 ± 5.6	Occupational status (%)	
Women (%)	56.8	Household	5.1
Born in Belgium (%)	96.3	Blue collar	5.3
Marital status (%)		White collar	67.9
Married	68.4	Unemployed	3.2
Widowed	1.6	Retired	17.5
Divorced	13.7	Career interruption	1.0
Single	7.6	Current bicycle transport level	
Cohabiting	8.6	No bicycle transport (%)	21.7
Education (%)		Bicycle transport min/wk (M ± SD)	147 ± 170
Primary	2.2	Living area	
Lower secondary	19.4	Urban (%)	15.4
Higher secondary	13.9	Suburban (%)	74.0
Tertiary	64.6	Rural (%)	10.6
BMI (M ± SD) (kg/m^2^)	25.2 ± 4.0		

*M* mean, *SD* standard deviation, *BMI* body mass index

**Fig. 2:**
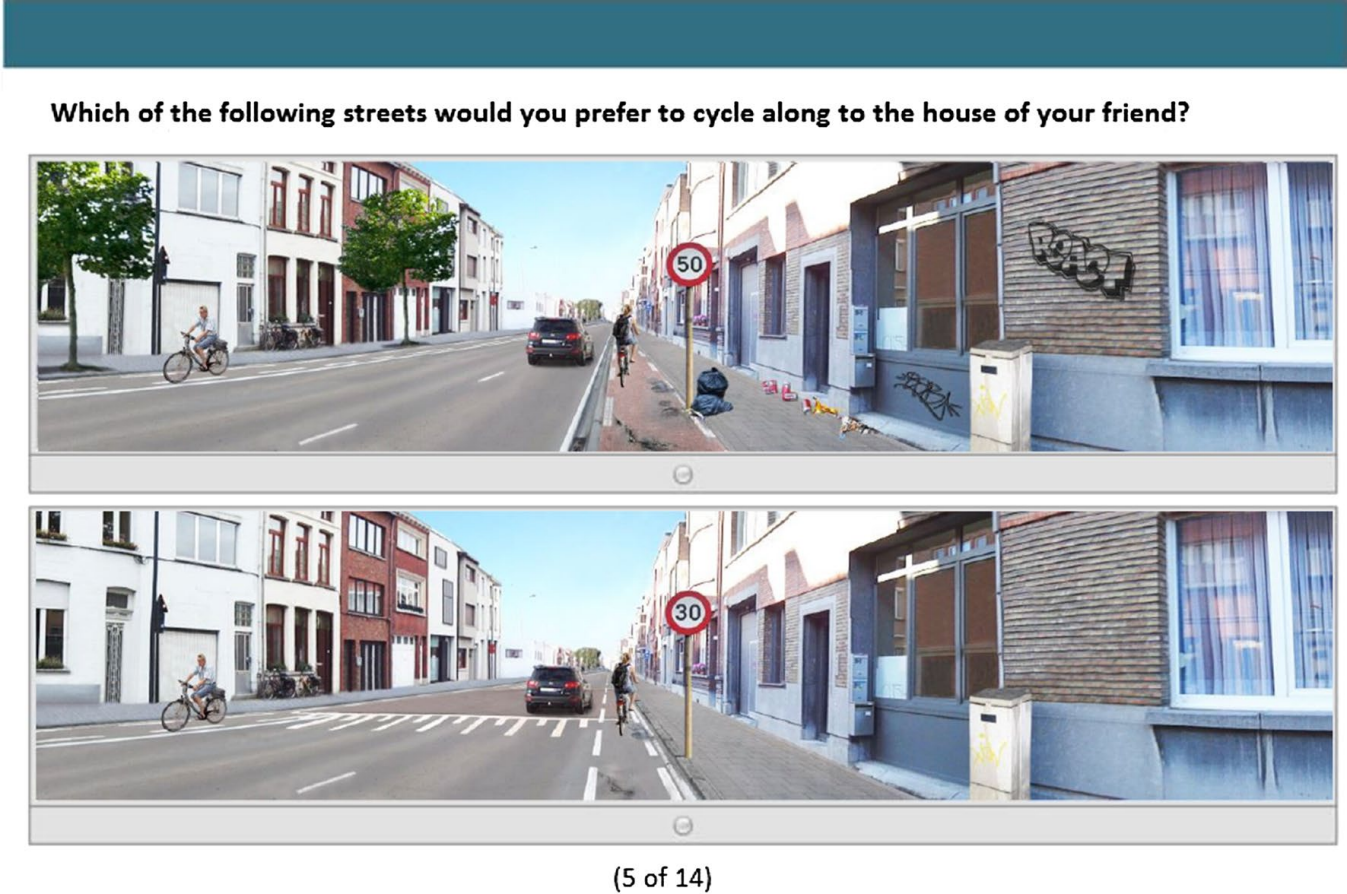
An example of a randomly assigned choice task used in the questionnaire

In the second part of the questionnaire, a choice based conjoint (CBC) method was used to implement a series of choice tasks with manipulated photographs, depicting two possible routes to cycle along. This CBC method is often used in marketing research and aims to identify the relative importance of various components of a product (micro-environmental factors in a street) in the decision process to pursuit the product (cycling for transport in that street) [61]. In this part, the following scenario was presented to the respondents: “Imagine yourself cycling to a friend’s home, located at 10 min cycling from your home, during daytime with perfect weather circumstances. For every task you will see two streets, we ask you to choose the street that you find most appealing to cycle along to that friend. Whichever route you choose, the distance to your friend is the same and all cycle paths are one-way. There is no right or wrong solution, we are only interested in which street you would prefer to cycle along.” Participants were first shown three examples and afterwards they received a set of 12 randomly assigned and two fixed choice tasks, which is a recommended quantity for such tasks [61, 62]. Figure 2 shows an example of a choice task. Since a full-profile design was used in the choice task, the two photographs in each randomly assigned choice task could differ in one to seven environmental factors [61]. The two fixed choice tasks were identical for all participants and were used to check if participants answered the choice tasks consistently. One respondent was deleted from the analysis as the response to both fixed tasks was not accurate in comparison with the other 1949 participants. We therefore believe that the respondent probably completed the questionnaire without attention.

A priori power analysis (power 0.80 and α = 0.05) was calculated by the following formula: *nta/c* > *500* (*n* = *number of participants; t* = *14: number of choice tasks; a* = *2: number of alternatives per task; c* = *18: the largest product of levels of any two factors*) [61]. This showed that a minimum of 322 subjects was needed when manipulating seven environmental factors in one photograph (with a maximum of six levels) and presenting 14 choice tasks to each participant. It was intended to reach at least three times more this number to allow possibly subgroup analysis.

To assess test–retest reliability of the choice tasks, we conducted a pilot study (n = 28) in which 14 fixed choice tasks were added to the questionnaire. These fixed choice tasks were identical for all participants. The same choice tasks were presented to the participants twice with a 1-week interval. Subsequently, it was examined whether participants chose the same street at both time points. The percentage of agreement for the 14 choice tasks ranged from 72 to 100 % (n = 28). These results indicated that our choice tasks are reliable, since an adequate level of agreement is generally considered to be 70 % [63].

### Analyses

Choice-based conjoint analysis (CBC) was used to analyze the data. First, the average relative importance of each environmental factor was calculated from the individual utility data gained from Hierarchical Bayes (HB) estimation using dummy coding. This analysis method has been suggested as the most appropriate method to analyze data gained from choice based conjoint [64]. Average relative importances indicate the influence of an environmental factor on the choice relating to the photograph choice task. These average importances are calculated by the difference in average part-worth utilities between the most and least preferred levels of a factor [61]. Average part-worth utilities represent the degree of preference given to a particular level of an environmental factor and are similar to a beta-value (β) obtained from linear regression analyses [61]. The greater the importance of an environmental factor, the greater the factor has an impact on the choice.

Second, the main effect of each level of each environmental factor on the street’s appeal for bicycle transport along the depicted environments was determined using the individual part-worth utilities gained from HB estimation. Average part-worth utilities were calculated and 95 % confidence intervals were determined to compare these part-worth utilities representing the degree of preference for the environmental factor level [61].

Third, interaction effects were also derived from part-worth utilities gained from the HB estimation and were selected using ‘CBC interaction search tool’ of the Sawtooth Software [65]. Separate models were constructed to analyze the interaction effects between different micro-environmental factors. These results were illustrated by graphs and tables in which the total utilities of the different streets were shown. Total utilities were calculated by the sum of the part-worth utilities and representing the degree of preference given to a photograph or for the environmental factors depicted in a street. A 95 % confidence interval was calculated to examine significance.

Last, given that different interaction effects were found with type of cycle path and that this factor is obvious most prominent, the relative importance of all other micro-environmental factors was calculated within each type of cycle path.

## Results

### Descriptive statistics

The sample consisted of 1950 participants ranging in age from 45 to 65 years: 56.8 % were women, 77.0 % were married or cohabiting, 64.6 % had followed tertiary education (college, university or postgraduate) and 17.5 % was retired (see Table 2). Mean age of the total sample was 54.3 years (SD = 5.6) and mean BMI was 25.2 kg/m^2^ (SD = 4.0). Approximately one fifth (21.7 %) of the adults did not cycle for transport in a usual week and the mean of the entire sample was 147 ± 170 min per week bicycle transport in a usual week.

### Relative importance of the micro-environmental factors

**Fig. 3:**
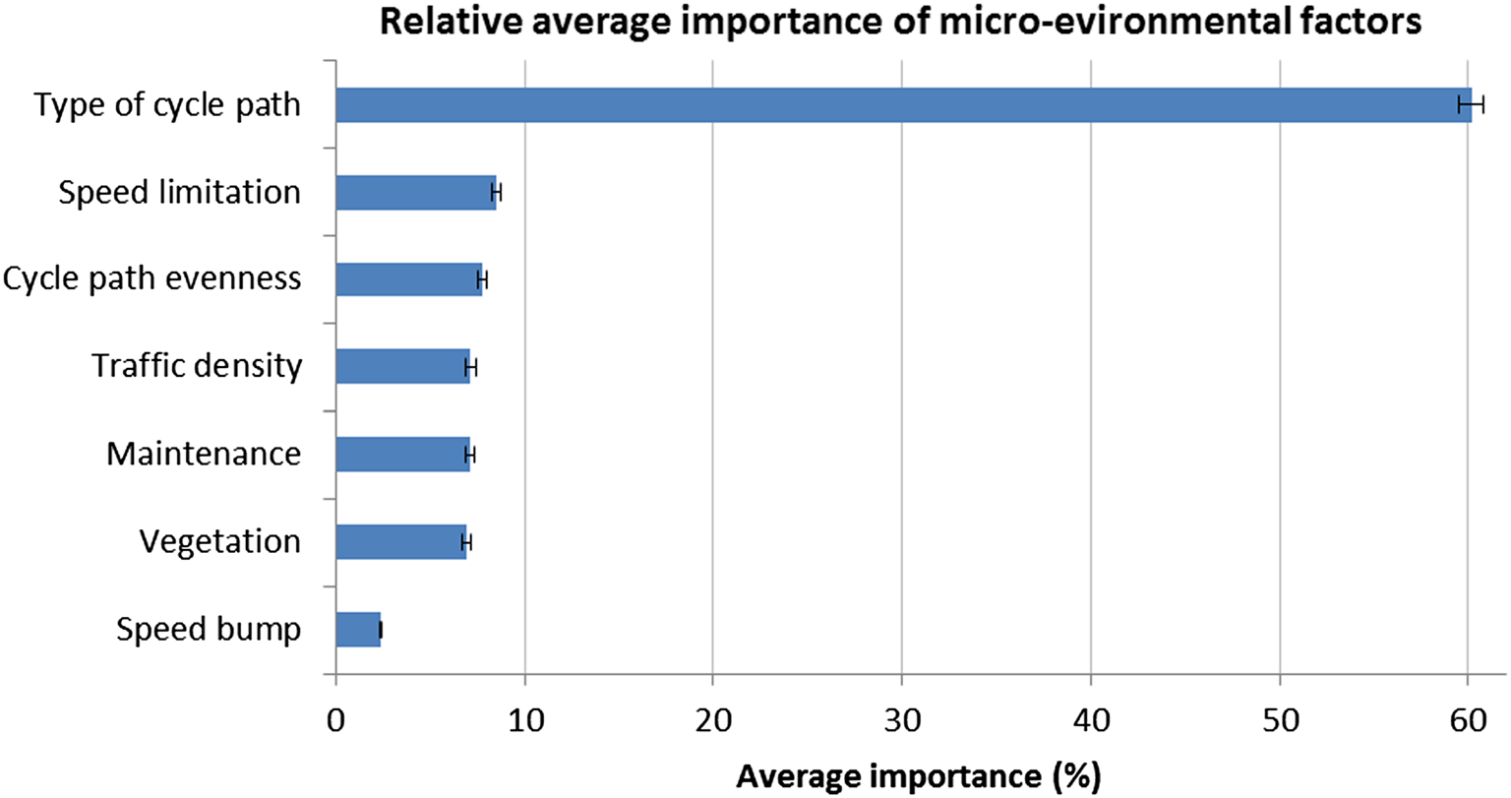
Relative importance of the micro-environmental factors

‘Type of cycle path’ (average importance = 60.14 ± 14.04 %; 95 % CI 59.48, 60.81) was by far the most important micro-environmental factor when choosing one out of two streets for bicycle transport (see Fig. 3). The second most important environmental factor was ‘speed limit’ (average importance = 8.50 ± 5.65 %; 95 % CI 8.25, 8.75) followed by ‘evenness of the cycle path surface’ (average importance = 7.76 ± 5.47 %; 95 % CI 7.52, 8.00). These factors were chosen over ‘traffic density’ (average importance = 7.14 ± 6.55 %; 95 % CI 6.85, 7.43), ‘general upkeep’ (average importance = 7.11 ± 5.53 %; 95 % CI 6.87, 7.36) and ‘vegetation’ (average importance = 6.96 ± 5.17 %; 95 % CI 6.73, 7.19) which did not significantly differ from each other. The presence of a ‘speed bump’ (average importance = 2.38 ± 1.86 %; 95 % CI 2.30, 2.47) was significantly less important than any other micro-environmental factor.

### Main effects of the environmental factors

**Fig. 4:**
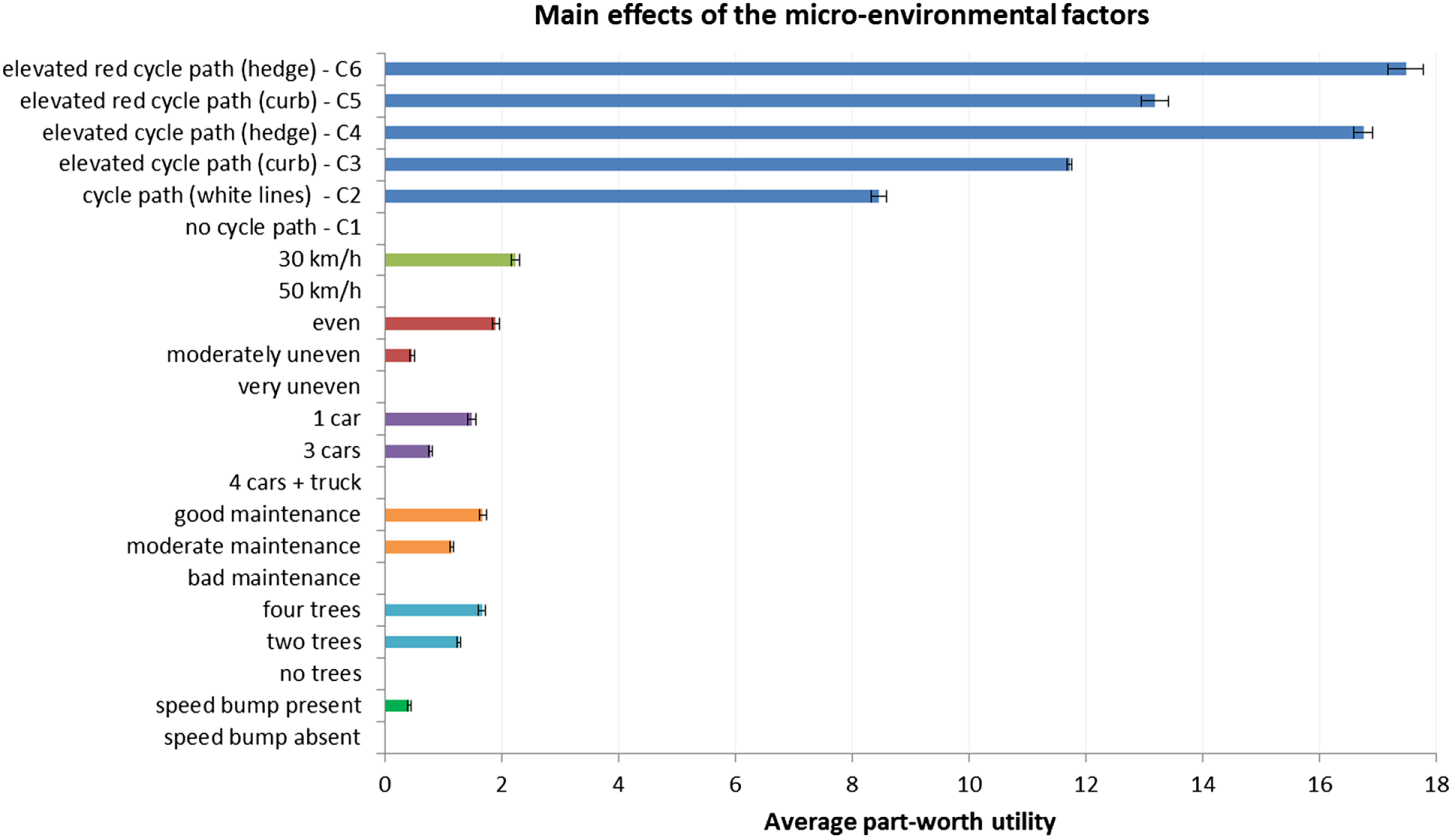
Main effects of the micro-environmental factors

**Fig. 5:**
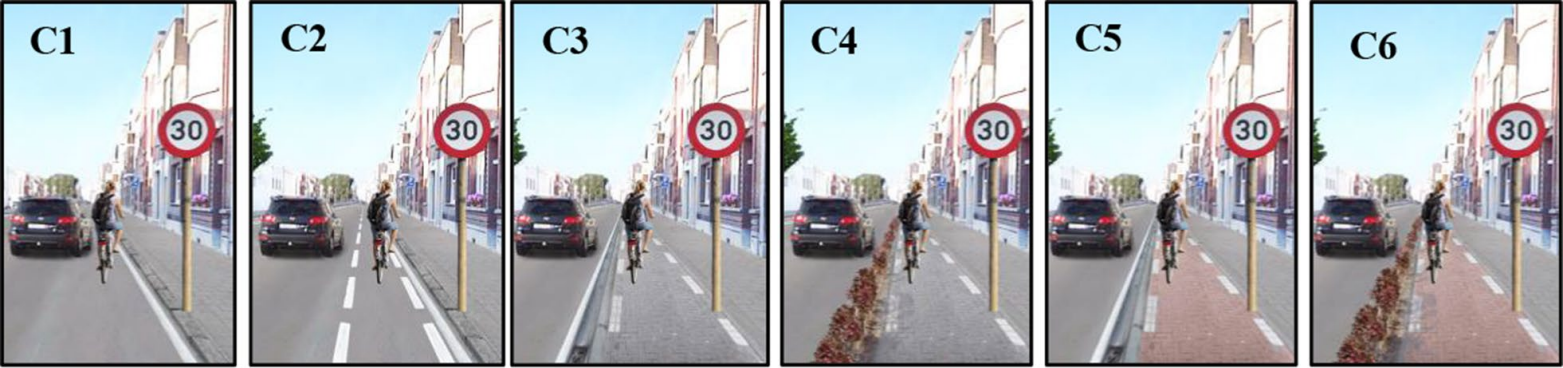
Different types of cycle paths manipulated in this study

Within each micro-environmental factor, all part-worth utilities from the different levels of each environmental factor significantly differed from each other (p < 0.05), with obvious preferences for the anticipated most attractive level over the intermediate and the anticipated unattractive level (see Fig. 4). For example, participants preferred an even cycle path surface (average part-worth utility = 1.90 ± 1.40; 95 % CI 1.84, 1.96) over a slightly uneven (average part-worth utility = 0.47 ± 1.04; 95 % CI 0.43, 0.52) and a very uneven cycle path surface (reference level); and they preferred a slightly uneven cycle path over a very uneven cycle path surface. One notable result was found for ‘type of cycle path’. A cycle path separated from traffic with a hedge and not separated from walking path by color was significantly more preferred (C4: average part-worth utility = 16.75 ± 3.64; 95 % CI 16.59, 16.91) than a cycle path separated from traffic with a curb and separated from walking path by color (C5: average part-worth utility = 13.18 ± 5.22; 95 % CI 12.95, 13.42). See Fig. 5 for an illustration of the different types of cycle paths manipulated in this study.

### Interaction effects

The combination of all possible interaction effects gave 21 possible interaction effects of which six were significant, namely ‘type of cycle path × speed limit’, ‘type of cycle path × vegetation’, ‘type of cycle path × evenness of the cycle path surface’, ‘type of cycle path × traffic density’, ‘speed bump × traffic density’, ‘vegetation × general upkeep’. The results of these interaction effects were illustrated by graphs and tables in which the total utilities of the different streets were shown. Total utilities represent the degree of preference and can be found in Additional files 1, 2, 3, 4, 5, 6.

**Fig. 6:**
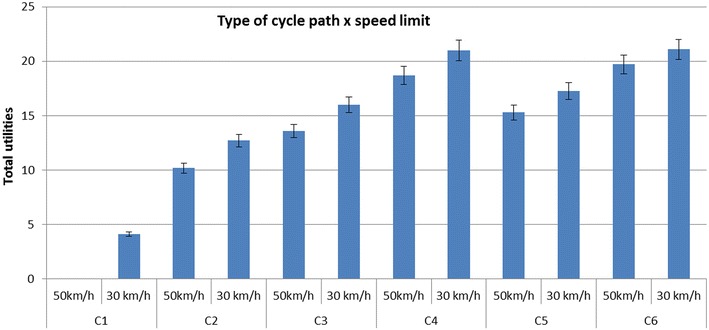
Interaction effect between cycle path type and speed limit. *C1* no cycle path; *C2* cycle path separated from traffic by marked white lines; *C3* cycle path separated from traffic with a curb, not separated from walking path by color; *C4* cycle path separated from traffic with a hedge, not separated from walking path by color; *C5* cycle path separated from traffic with a curb, separated from walking path by color; *C6* cycle path separated from traffic with a hedge, separated from walking path by color

The significant interaction effect between ‘type of cycle path’ and ‘speed limit’ (Chi square = 16.87; p = 0.005) shows that the effect of speed limit has the greatest impact on the street’s appeal for bicycle transport when there was no cycle path (C1) (see Fig. 6; Table A.1 in Additional file 1). Adjusting the speed limit from 50 to 30 km/h along all different cycle paths had a significant effect, except for the most preferred cycle path. The effect of speed limit did not provide a significant increase on the street’s appeal for bicycle transport when the cycle path was separated from traffic with a hedge and separated from walking path by color (C6).

The significant interaction effect between ‘type of cycle path’ and ‘vegetation’ (Chi square = 27.78; p = 0.002) shows that the effect of vegetation was significant in all different types of cycle paths (see Additional file 2). The direction of the effects did not differ, only the magnitude of the effect did. For instance, the greatest effect of vegetation (from zero to four trees) was found when there was no cycle path provided on the street, compared to all types of cycle path.

Similar results were found for the interaction effect between ‘type of cycle path’ and ‘traffic density’ (Chi square = 19.01; p < 0.001). The effect of traffic density was significant for all different types of cycle paths in the expected direction, only the strength of the effect differed across the different cycle paths (see Additional file 3). The greatest effect of traffic density on the street’s appeal for bicycle transport was found when there was no cycle path.

The significant interaction effect between ‘type of cycle path’ and ‘evenness of the cycle path surface’ (Chi square = 44.94; p = 0.040) showed that the greatest effect of evenness of the cycle path surface (from very uneven or moderately uneven to an even cycle path surface) was found with cycle paths where a separation with motorized traffic by a curb is provided (see Additional file 4). The greatest effects from a very uneven to an even cycle path surface on the street’s appeal for bicycle transport was found with a cycle path separated from traffic with a curb and separated from walking path by color (C5). Additionally, the greatest effect of evenness from a moderately uneven to an even cycle path surface on the street’s appeal was found with a cycle path separated from traffic with a curb and not separated from walking path by color (C3).

There was also a significant interaction effect between ‘speed bump’ and ‘traffic density’ (Chi square = 9.71; p = 0.008). The effect of a speed bump (installing a speed bump on the street) on the street’s appeal for bicycle transport, was greater when the traffic density was lower (reducing the number of cars to the intermediate or lowest level) (see Additional file 5).

Finally, the significant interaction effect between ‘vegetation’ and ‘general upkeep’ (Chi square = 10.19; p = 0.040) showed that depending on the number of trees another effect of general upkeep was found (see Additional file 6). The effect of general upkeep from moderate to good upkeep was greater if there were no trees present in the environment. The effect of general upkeep from bad to good upkeep was greater in an environment with two trees and the effect between bad and moderate upkeep was greater in an environment with four trees.

### Relative importance of the micro-environmental factors within different cycle paths

Given that several interaction effects were found with ‘cycle path type’ and it appeared to be by far the most important micro-environmental factor in making choices among different street alternatives, the relative importance of all other environmental factors within each type of cycle path was defined. It is useful to determine which priority must be given in adapting the environment if a community does not have the ability to build a desired cycle path.

**Fig. 7:**
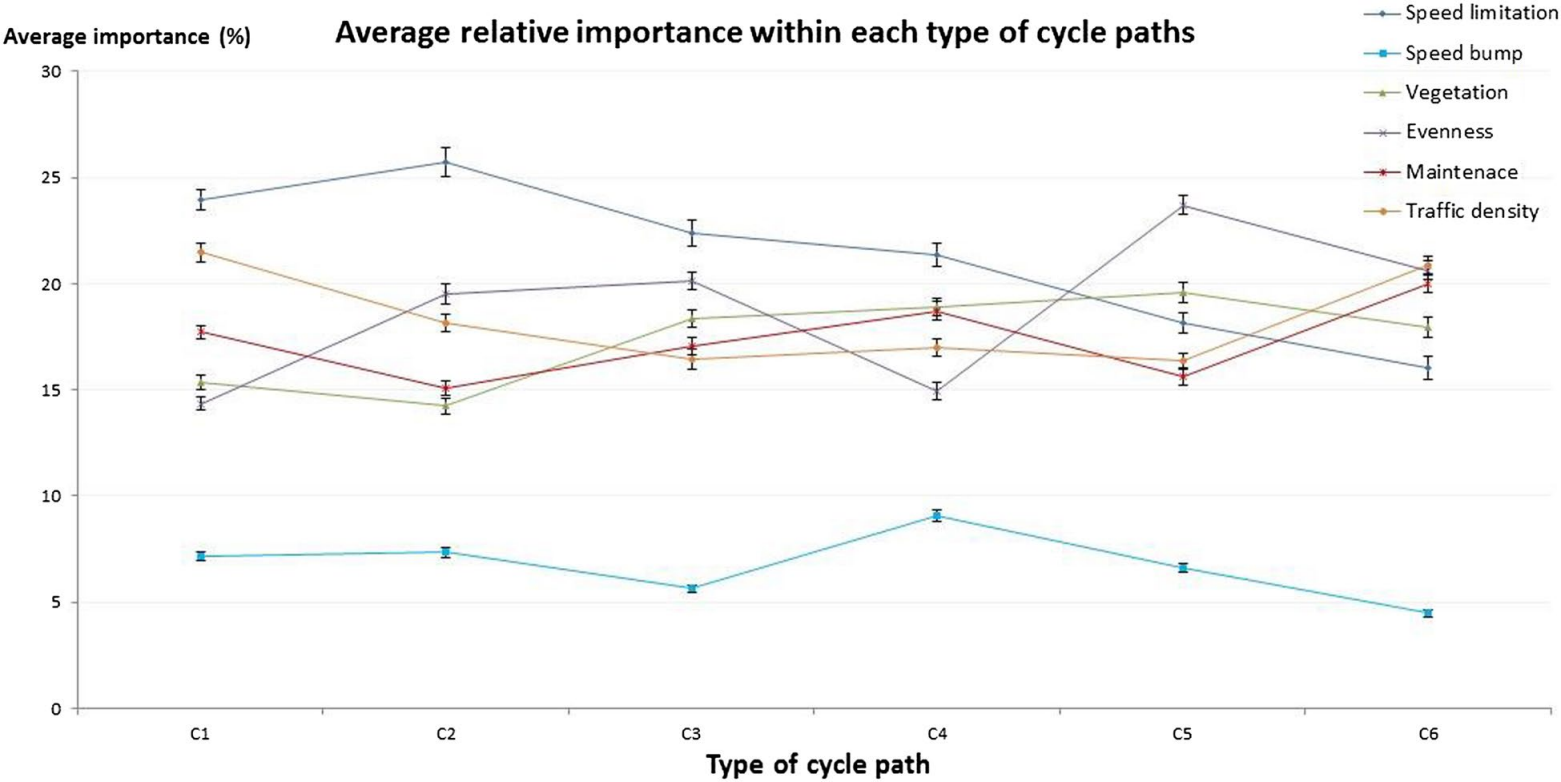
Average relative importance of the six environmental factors within the different cycle path types. *C1* no cycle path; *C2* cycle path separated from traffic by marked white lines; *C3* cycle path separated from traffic with a curb, not separated from walking path by color; *C4* cycle path separated from traffic with a hedge, not separated from walking path by color; *C5* cycle path separated from traffic with a curb, separated from walking path by color; *C6* cycle path separated from traffic with a hedge, separated from walking path by color

In Fig. 7, the relative importance of the remaining six environmental factors is presented for each type of cycle path. The results showed that modifying the speed limit was the most important environmental factor in situations where there was no cycle path (C1), no elevated cycle path (C2) or no cycle path with separations at both sides (C3 and C4). When there was no cycle path present in the environment (C1), the effect of speed limit (average part-worth utility = 23.97 ± 10.96 %; 95 % CI 23.48, 24.45) and traffic density (average part-worth utility = 21.46 ± 9.75 %; 95 % CI 21.03, 21.89) created the largest impact on the street’s appeal for bicycle transport. Furthermore, with increasing separation (going from C1 to C6), speed limit appeared to become less important. In situations where the most preferred type of cycle path was already present (C6: elevated cycle path, separated from motorized traffic with a hedge and separated from the walking path by color), the first three micro-environmental factors did not significantly differ from each other: traffic density (average part-worth utility = 20.87 ± 9.93 %; 95 % CI 20.43, 21.31), evenness of the cycle path surface (average part-worth utility = 20.61 ± 9.98 %; 95 % CI 20.17, 21.05) and general upkeep (average part-worth utility = 20.01 ± 9.77 %; 95 % CI 19.58, 20.44). Moreover, the effect of speed limit was significantly lower when the most preferred cycle path was present (C6) compared to situations when less preferred cycle paths were present.

## Discussion

We identified the micro-environmental factors that should get priority when adapting the micro-environment to increase the street’s appeal for middle-aged adults’ bicycle transport. In addition, we investigated the interaction effects between different micro-environmental factors. The current study proved that the ‘type of the cycle path’ appeared to be the most important micro-environmental factor affecting the street’s appeal for adults’ bicycle transport under optimal conditions in terms of trip length and trip objective. A cycle path separated from traffic with a hedge was significantly more preferred than a cycle path separated from traffic with a curb, regardless of the separation from walking path by color. Previous research already showed a positive outcome of having a good separation between cyclists and motorized traffic on bicycle transport but did not focus on the relative importance of different types of ‘separations’ [49, 66, 67]. One of these studies indicated that research should also focus on the different designs to separate cyclists from cars [67]. The present study presented an initial possibility to investigate different gradation levels for possible separation between motorized traffic and cycle path (i.e. marked white lines—curb—hedge). A remarkable result in the present study was the large effect on the street’s appeal of the most preferred type of separation, a small hedge. As a small hedge will not provide complete protection for cyclists from cars, it will merely be the perception of a separation that apparently makes them feel safer. Increased traffic safety or only the perception of it will be of great importance. This corresponds to recent findings, indicating that implementing measures to improve cyclists’ safety from cars could increase cycling [66]. The current study showed that adapting the cycle path should get priority over other micro-environmental factors, such as speed limit, speed bump, vegetation, evenness of the cycle path surface and general upkeep. Even when it is not possible to actually separate cyclists from motorized traffic with a hedge, the presence of a curb or an indication by marked white lines may stimulate bicycle transport. An additional separation between cycle path and walking path by color will increase the street’s appeal even more, but much less pronounced in comparison with the benefit obtained by a suitable separation with motorized traffic.

Changing the type of cycle path might not be possible in all situations (e.g. financial or space constraints). Therefore, we also investigated the relative importance of the environmental factors within each type of cycle path which has not been studied previously. When there are no possibilities to provide a separation between cycle path and motorized traffic, adjusting the speed of the traffic from 50 km/h to 30 km/h may ensure an increase in the street’s appeal for bicycle transport. Furthermore, traffic density was found to be the second most important environmental factor to adapt when there is no cycle path in the street. Similar results were found for the interaction effects; decreasing the traffic speed or traffic density has a larger effect on the street’s appeal for bicycle transport when there is no cycle path provided in the street compared to situations where other cycle paths are present. On the other hand, modifying the speed limit from 50 to 30 km/h has no additional effect on the street’s appeal when the most preferred cycle path is present. These results indicate that in situations where there is no cycle path provided, micro-environmental factors associated to traffic-related safety appear to be most prominent. These findings should be communicated to policies at national and subnational level encouraging bicycle transport. The first priority when executing environmental interventions is the provision of a cycle path. If this adjustment is not practically feasible, micro-environmental factors related to safety (i.e., speed limit, traffic density) may be more effective in promoting bicycle transport than micro-environmental factors related to comfort (i.e. evenness of the cycle path surface) or aesthetic (i.e. vegetation, general upkeep). The importance of traffic safety regarding bicycle transport has also been mentioned in the literature [10, 49, 67]. The study of Fraser and Lock [49] noticed that when we want to create safe environments, we need to improve our research on the built environment prioritizing the needs of cyclists, including the evaluation of both rates of physical activity and road injury [49].

Furthermore, when a more separated cycle path (going from C1 to C6) is provided, micro-environmental factors related to comfort (i.e. evenness of the cycle path surface) or aesthetic (i.e. vegetation, general upkeep) appeared to become more important. For example, the effect of evenness obtained from the interaction analysis showed that increasing the evenness of the cycle path surface has the greatest effect on the street’s appeal when a cycle path is separated from traffic with a curb. Improving the evenness of the cycle path surface could increase the street’s appeal for bicycle transport even more when there is already a separation by means of a curb present. Moreover, when the most preferred cycle path is present (separated from traffic with a hedge and separated from walking path by color), the relative importance of the other environmental factors became more similar. In this situation, it does not matter which of the three micro-environmental factors (‘traffic density’, ‘evenness of the cycle path surface’ or ‘general upkeep’) will be modified first. They may achieve the same effect on bicycle transport because these factors did not significantly differ in importance from each other.

The effect of vegetation (from the lowest or intermediate to the anticipated most attractive level) on the street’s appeal for bicycle transport was the greatest when there was no cycle path provided in the street. But on the other hand, we also know that when there is no cycle path provided, other micro-environmental factors turn out to be more important than vegetation. Nevertheless, the presence of vegetation may contribute to the street’s appeal as second important factor in situations with a cycle path separated from traffic with a hedge (C4) or a cycle path separated from traffic with a curb and separated from walking path by color (C5).

Although, speed bump is the least preferred micro-environmental factor of all seven, the effect of the presence of a speed bump can be enhanced by reducing traffic density. Providing the street of a speed bump should not get priority over the other environmental factors, but a recommendation to the transport policies could be that adapting both factors together (speed bump and traffic density) is better than just focusing on installing a speed bump. A possible explanation for this effect could be found with the help of qualitative data from a recent mixed-method study [48], in which participants argued that the presence of a speed bump indirectly shows that many cars drive in the street.

The main strength of the current study was the used methodology (i.e. the choice based conjoint method using manipulated photographs) to answer the research questions. In real life, when people choose a route to cycle along to go to a place, they have to choose between combinations of factors. For example, people could make a decision by considering multiple factors such as limited speed of the cars, an even cycle path, some green along the route. Therefore, it is important to identify which factors are more important than others in such complex decisional contexts in order to understand how to create more encouraging cycling environments. The CBC method using manipulated photographs could identify the relative importance of micro-environmental factors in a street’s appeal to cycle for transport [61]. This methodology allows studying the effects of environmental changes (manipulations) under controlled conditions, i.e. controlling the variation within and between the manipulated micro-environmental factors. The controlled manipulations of micro-environmental factors in the photographs are a cost-effective approach and could be used to experimentally find out which factors affect a street’s appeal for bicycle transport under optimal conditions in terms of trip length and trip objective. Findings obtained from this study could provide practical guidelines for environmental interventions focusing on adapting micro-environmental factors to create more supportive environments for bicycle transport. From a previous study we know that these findings are not only valid for the street context depicted in the photographs of current study (i.e. a typical street environment in a semi-urban (300–600 inhabitants/km^2^) Belgian municipality [57]), but most likely also for other street contexts (i.e. an environment with low building density and single land use or an environment with high building density and mixed land use) [59]. To our knowledge, this is the first study that creates an order of importance or hierarchy of the different micro-environmental factors. Furthermore, also interaction effects between different environmental factors were examined. Finally, by disseminating the research through the web, a very large sample was reached. However, this method also involved some disadvantages. Participants with a tertiary education (64.6 %) and a white collar occupation status (67.9 %) were over-represented in our study compared with the statistics of the Flemish population [68]; where 28.1 % has a tertiary degree and the majority of the adults has a blue collar occupation. With our research, we have reached mainly highly educated people. Future research needs to establish whether these findings can be generalized to the entire Flemish population of mid-aged adults. Another limitation of current study is the two-dimensional character or the lack of movement/noise in the photograph environments. This can be overcome by using three-dimensional methods like manipulating computer-generated virtual walkthrough environments [69]. Nevertheless, using such methods is very expensive and only small samples can be reached. Finally, the most important weakness is that the current study did not assess effects on actual cycling behavior, but only on the street’s appeal for bicycle transport. Consequently, these findings need to be confirmed by on-site research.

Some suggestions for future research can be made. A first suggestion is to compare our findings with results of other age groups. In the current study, only middle-aged adults between 45 and 65 years old were included to assess the viewpoint of the adult population. Besides this, also the viewpoint of younger adults assessing the environment in the perspective of their child is an important contributor as well as the viewpoint of older adults. Since, interventions targeting the built environment to encourage active transport, can reach a large proportion of the population [15], it is important to determine whether the same micro-environmental factors are important for different age-groups. Secondly, the current study fixed both trip objective and trip length. It would be interesting for future research to investigate the role of these environmental factors in relation to the preferred cycling route. Thirdly, integrating the role of socio-environmental factors (e.g. neighborhood safety) might enrich future studies’ inputs and results. Finally, future research should also investigate the moderating effects of socio-demographics, psychosocial correlates and bicycle use on the relationship between the micro-environment and bicycle transport.

## Conclusions

To our knowledge, this is the first study that creates an order of importance or hierarchy of relevant micro-environmental factors. Furthermore, also interaction effects between different environmental factors were examined as well as the relative importance of environmental factors within a particular micro-environmental factor. Providing streets with a cycle path separated from motorized traffic seems to be the best strategy to increase the street’s appeal for adults’ bicycle transport. A cycle path marked by white lines can already contribute to this, but a separation between cycle path and motorized traffic by means of a curb or a hedge appeared to be preferred. An additional separation with the walking path by color would increase the street’s appeal for bicycle transport even more. If this adjustment is not practically feasible, micro-environmental factors related to safety (i.e., speed limit, traffic density) may be more effective in promoting bicycle transport than micro-environmental factors related to comfort (i.e. evenness of the cycle path surface) or aesthetic (i.e. vegetation, general upkeep). Furthermore, when a more separated cycle path is provided, micro-environmental factors related to comfort (i.e. evenness of the cycle path surface) or aesthetic (i.e. vegetation, general upkeep) appeared to increase in importance. Findings obtained from this research could provide advice to physical environmental interventions about which environmental factors should get priority to modify in different environmental situations.

## Additional files

10.1186/s12942-016-0058-4 Interaction effect between cycle path type and speed limit.
10.1186/s12942-016-0058-4 Interaction effect between cycle path type and vegetation.
10.1186/s12942-016-0058-4 Interaction effect between cycle path type and traffic density.
10.1186/s12942-016-0058-4 Interaction effect between cycle path type and evenness.
10.1186/s12942-016-0058-4 Interaction effect between speed bump and traffic density.
10.1186/s12942-016-0058-4 Interaction effect between vegetation and general upkeep.

## Abbreviations

C1: no cycle path
C2: cycle path, separated from traffic by marked white lines
C3: cycle path, separated from traffic with a curb, not separated from walking path by color
C4: cycle path separated from traffic with a hedge, not separated from walking path by color
C5: cycle path separated from traffic with a curb, separated from walking path by color
C6: cycle path separated from traffic with a hedge, separated from walking path by color

## References

[1] Rudinger G, Donaghy K, Poppelreuter S (2006). Societal trends, mobility behaviour and sustainable transport in Europe and North America. Eur J Transp Infrastruct Res..

[2] Vlaamse overheid Departement Mobiliteit en Openbare Werken. Onderzoek Verplaatsingsgedrag Vlaanderen 4. Brussel.

[3] Wanner M, Götschi T, Martin-Diener E, Kahlmeier S, Martin BW (2012). Active transport, physical activity, and body weight in adults: a systematic review. Am J Prev Med.

[4] World Health Organization. Global Recommendations on Physical Activity for Health. 2010. p. 1–60. http://apps.who.int/iris/bitstream/10665/44399/1/9789241599979_eng.pdf. Accessed 25 Aug 2016. 26180873

[5] Oja P, Titze S, Bauman A, de Geus B, Krenn P, Reger-Nash B, Kohlberger T (2011). Health benefits of cycling: a systematic review. Scand J Med Sci Sports.

[6] Kelly P, Kahlmeier S, Götschi T, Orsini N, Richards J, Roberts N, Scarborough P, Foster C (2014). Systematic review and meta-analysis of reduction in all-cause mortality from walking and cycling and shape of dose response relationship. Int J Behav Nutr Phys Act.

[7] Rissel CE (2009). Active travel: a climate change mitigation strategy with co-benefits for health. N S W Public Health Bull.

[8] Woodcock J, Edwards P, Tonne C, Armstrong BG, Ashiru O, Banister D, Beevers S, Chalabi Z, Chowdhury Z, Cohen A, Franco OH, Haines A, Hickman R, Lindsay G, Mittal I, Mohan D, Tiwari G, Woodward A, Roberts I (2009). Public health benefits of strategies to reduce greenhouse-gas emissions: urban land transport. Lancet.

[9] Departement of Health, Physical Activity. Health improvement and prevention: at least five a week. 2004. p. 1–128. http://www.bhfactive.org.uk/sites/Exercise-Referral-Toolkit/downloads/resources/cmos-report-at-least-five-a-week.pdf. Accessed 25 Aug 2016.

[10] Pucher J, Buehler R, Bassett DR, Dannenberg AL (2010). Walking and cycling to health: a comparative analysis of city, state, and international data. Am J Public Health.

[11] Rabl A, de Nazelle A (2012). Benefits of shift from car to active transport. Transp Policy.

[12] Active Transport. https://secure.ausport.gov.au/clearinghouse/knowledge_base/organised_sport/sport_and_government_policy_objectives/active_transport.

[13] de Hartog JJ, Boogaard H, Nijland H, Hoek G (2010). Do the health benefits of cycling outweigh the risks?. Environ Health Perspect.

[14] Rojas-Rueda D, de Nazelle A, Tainio M, Nieuwenhuijsen MJ (2011). The health risks and benefits of cycling in urban environments compared with car use: health impact assessment study. BMJ.

[15] World Health Organization. Interventions on diet and physical activity: what works (summary report). 2009. p. 1–48. http://www.who.int/dietphysicalactivity/summary-report-09.pdf. Accessed 25 Aug 2016. 24432437

[16] Vandenbulcke G, Thomas I, de Geus B, Degraeuwe B, Torfs R, Meeusen R, Panis LI (2009). Mapping bicycle use and the risk of accidents for commuters who cycle to work in Belgium. Transp Policy.

[17] Jongeneel-Grimen B, Busschers W, Droomers M, van Oers HA, Stronks K, Kunst AE (2013). Change in neighborhood traffic safety: does it matter in terms of physical activity?. PLoS ONE.

[18] Sallis JF, Cervero RB, Ascher W, Henderson KA, Kraft MK, Kerr J (2006). An ecological approach to creating active living communities. Annu Rev Public Health.

[19] Gaffron P (2003). The implementation of walking and cycling policies in British local authorities. Transp Policy.

[20] Pucher J, Dill J, Handy S (2010). Infrastructure, programs, and policies to increase bicycling: an international review. Prev Med.

[21] Buehler R, Pucher J. Walking and cycling in Western Europe and the United States. TR NEWS (280) 2012.

[22] Commission of the European Communities (2007). GREEN PAPER towards a new culture for urban mobility.

[23] World Health Organization. Global Status report on noncommunicable diseases. 2014. p. 1–302. http://apps.who.int/iris/bitstream/10665/148114/1/9789241564854_eng.pdf. Accessed 25 Aug 2016.

[24] Pucher J, Buehler R (2007). Cycling for everyone: lessons from Europe. J Transp Res Board.

[25] Swinburn B, Egger G, Raza F (1999). Dissecting obesogenic environments: the development and application of a framework for identifying and prioritizing environmental interventions for obesity. Prev Med..

[26] Cain KL, Millstein RA, Sallis JF, Conway TL, Gavand KA, Frank LD, Saelens BE, Geremia CM, Chapman J, Adams MA, Glanz K, King AC (2014). Contribution of streetscape audits to explanation of physical activity in four age groups based on the Microscale Audit of Pedestrian Streetscapes (MAPS). Soc Sci Med (1982).

[27] Van Holle V, Deforche B, Van Cauwenberg J, Goubert L, Maes L, Van de Weghe N, De Bourdeaudhuij I (2012). Relationship between the physical environment and different domains of physical activity in European adults: a systematic review. BMC Public Health.

[28] Saelens BE, Sallis JF, Frank LD (2003). Environmental correlates of walking and cycling: findings from the transportation, urban design, and planning literatures. Ann Behav Med.

[29] Van Dyck D, Cerin E, Conway TL, De Bourdeaudhuij I, Owen N, Kerr J, Cardon G, Frank LD, Saelens BE, Sallis JF (2012). Perceived neighborhood environmental attributes associated with adults’ transport-related walking and cycling: findings from the USA, Australia and Belgium. Int J Behav Nutr Phys Act.

[30] McCormack GR, Shiell A (2011). In search of causality: a systematic review of the relationship between the built environment and physical activity among adults. Int J Behav Nutr Phys Act.

[31] Foster CE, Panter JR, Wareham NJ (2011). Assessing the impact of road traffic on cycling for leisure and cycling to work. Int J Behav Nutr Phys Act.

[32] Wendel-Vos W, Droomers M, Kremers S, Brug J, van Lenthe F (2007). Potential environmental determinants of physical activity in adults: a systematic review. Obes Rev.

[33] McCormack G, Giles-Corti B, Lange A, Smith T, Martin K, Pikora TJ (2004). An update of recent evidence of the relationship between objective and self-report measures of the physical environment and physical activity behaviours. J Sci Med Sport.

[34] Bauman AE, Reis RS, Sallis JF, Wells JC, Loos RJF, Martin BW (2012). Correlates of physical activity: why are some people physically active and others not?. Lancet.

[35] Van Holle V, Van Cauwenberg J, Deforche B, Goubert L, Maes L, Nasar J, Van de Weghe N, Salmon J, De Bourdeaudhuij I (2014). Environmental invitingness for transport-related cycling in middle-aged adults: a proof of concept study using photographs. Transp Res Part A Policy Pract.

[36] Vandenbulcke G, Dujardin C, Thomas I, De Geus B, Degraeuwe B, Meeusen R, Panis LI (2011). Cycle commuting in Belgium: spatial determinants and “re-cycling” strategies. Transp Res Part A Policy Pract.

[37] de Geus B, De Bourdeaudhuij I, Jannes C, Meeusen R (2008). Psychosocial and environmental factors associated with cycling for transport among a working population. Health Educ Res.

[38] Titze S, Stronegger WJ, Janschitz S, Oja P (2007). Environmental, social, and personal correlates of cycling for transportation in a student population. J Phys Act Health..

[39] Titze S, Giles-Corti B, Knuiman MW, Pikora TJ, Timperio A, Bull FC, Van Niel K (2010). Associations between intrapersonal and neighborhood environmental characteristics and cycling for transport and recreation in adults: baseline results from the RESIDE study. J Phys Act Health.

[40] Van Dyck D, Cardon G, Deforche B, Giles-Corti B, Sallis JF, Owen N, De Bourdeaudhuij I (2011). Environmental and psychosocial correlates of accelerometer-assessed and self-reported physical activity in Belgian adults. Int J Behav Med.

[41] Parkin J, Wardman M, Page M (2008). Estimation of the determinants of bicycle mode share for the journey to work using census data. Transportation.

[42] Lee C, Moudon AV (2008). Neighbourhood design and physical activity. Build Res Inf..

[43] Zlot AI, Schmid TL (2005). Relationships among community characteristics and walking and bicycling for transportation or recreation. Am J Health Promot.

[44] Wendel-vos W, Schuit J, De Niet R, Boshuizen HC, Saris WHM, Kromhout D (2004). Factors of the physical environment associated with walking and bicycling. Med Sci Sports Exerc..

[45] Kondo K, Su ÆJ, Kiyoshi LÆ, Yusuke KÆ, Takagi H, Sunagawa ÆH, Akabayashi ÆA (2009). Association between daily physical activity and neighborhood environments. Environ Health Prev Med..

[46] Caulfield B, Brick E, Thérèse O (2012). Determining bicycle infrastructure preferences—a case study of Dublin. Transp Res Part D.

[47] Evenson KR, Herring AH, Huston SL (2005). Evaluating change in physical activity with the building of a multi-use trail. Am J Prev Med..

[48] Mertens L, Van Holle V, De Bourdeaudhuij I, Deforche B, Salmon J, Nasar J, Van de Weghe N, Van Dyck D, Van Cauwenberg J (2014). The effect of changing micro-scale physical environmental factors on an environment’s invitingness for transportation cycling in adults: an exploratory study using manipulated photographs. Int J Behav Nutr Phys Act.

[49] Fraser SDS, Lock K (2010). Cycling for transport and public health: a systematic review of the effect of the environment on cycling. Eur J Public Health.

[50] Carpiano RM (2009). Come take a walk with me: the “go-along” interview as a novel method for studying the implications of place for health and well-being. Health Place.

[51] Spittaels H, Foster C, Oppert J-M, Rutter H, Oja P, Sjöström M, De Bourdeaudhuij I (2009). Assessment of environmental correlates of physical activity: development of a European questionnaire. Int J Behav Nutr Phys Act.

[52] Sallis JF, Bowles HR, Bauman A, Ainsworth BE, Bull FC, Craig CL, Sjöström M, De Bourdeaudhuij I, Lefevre J, Matsudo V, Matsudo S, Macfarlane DJ, Gomez LF, Inoue S, Murase N, Volbekiene V, McLean G, Carr H, Heggebo LK, Tomten H, Bergman P (2009). Neighborhood environments and physical activity among adults in 11 countries. Am J Prev Med.

[53] Ferdinand AO, Sen B, Rahurkar S, Engler S, Menachemi N (2012). The relationship between built environments and physical activity: a systematic review. Am J Public Health..

[54] Nasar JL (2008). Assessing perceptions of environments for active living. Am J Prev Med.

[55] Wells NM, Ashdown SP, Davies EHS, Cowett FD, Yang Y (2007). Environment, design, and obesity: opportunities for interdisciplinary collaborative research. Environ Behav.

[56] Adobe Systems Incorporated: Adobe Photoshop CC. 2013. p. 1–87. http://wwwimages.adobe.com/content/dam/Adobe/en/devnet/photoshop/pdfs/photoshop-cc-scripting-guide.pdf. Accessed 25 Aug 2016.

[57] Lenders S, Lauwers L, Vervloet D, Kerselaers E. Afbakening van het Vlaamse platteland, een statistische analyse. 2006. p. 1–63. http://www2.vlaanderen.be/landbouw/downloads/volt/38.pdf. Accessed 25 Aug 2016.

[58] Pucher J, Buehler R (2008). Making cycling irresistible: lessons from the Netherlands, Denmark and Germany. Transp Rev.

[59] Mertens L, Van Cauwenberg J, Ghekiere A, Van Holle V, De Bourdeaudhuij I, Deforche B, Nasar J, Van de Weghe N, Van Dyck D. Does the effect of micro-environmental factors on a street’s appeal for adults' bicycle transport vary across different macro-environments? An experimental study. PLoS One 2015;10:1–17. 10.1371/journal.pone.0136715 PMC4552783 26317754

[60] Craig CL, Marshall AL, Sjöström M, Bauman AE, Booth ML, Ainsworth BE, Pratt M, Ekelund U, Yngve A, Sallis JF, Oja P (2003). International physical activity questionnaire: 12-country reliability and validity. Med Sci Sports Exerc.

[61] Orme BK (2009). Getting started with conjoint analysis: strategies for product design and pricing research.

[62] Sawtooth Software Inc. The CBC system for choice-based conjoint analysis. 2013. p. 1–27. https://sawtoothsoftware.com/download/techpap/cbctech.pdf. Accessed 25 Aug 2016.

[63] Multon KD. Interrater reliability. In: Salkind NJ, editor. Encyclopedia research design. Thousand Oaks, CA: Sage; 2012. p. 627–629. doi:10.4135/9781412961288.n194. http://methods.sagepub.com/reference/encyc-of-research-design/n194.xml. Accessed 25 Aug 2016.

[64] Allenby GM, Arora N, Ginter JL (1998). On the heterogeneity of demand. J Mark Res..

[65] Interaction Search Tool. https://www.sawtoothsoftware.com/help/issues/ssiweb/online_help/index.html?interaction_search_tool.htm.

[66] Sallis JF, Conway TL, Dillon LI, Frank LD, Adams MA, Cain KL, Saelens BE (2013). Environmental and demographic correlates of bicycling. Prev Med.

[67] Winters M, Davidson G, Kao D, Teschke K (2010). Motivators and deterrents of bicycling: comparing influences on decisions to ride. Transportation.

[68] Statistics Belgium. http://statbel.fgov.be/.

[69] Cubukcu E, Nasar JL (2005). Influence of physical characteristics of routes on distance cognition in virtual environments. Environ Plan.

