# Supplementary material for: Which environmental factors most strongly influence a street’s appeal for bicycle transport among adults? A conjoint study using manipulated photographs
Source: Int J Health Geogr. 2016 Sep 1;15(1):31. doi: 10.1186/s12942-016-0058-4 (PMC5007833; doi:10.1186/s12942-016-0058-4)
Supplement: Supplementary file 1 — 10.1186/s12942-016-0058-4 Interaction effect between cycle path type and speed limit. [file 12942_2016_58_MOESM1_ESM.pdf]

Additional file 1 - Interaction effect between cycle path type and speed limit

Table A.1. Interaction effect between type of cycle path and speed limit (chi-square= 16.87; p=0.005)

	C1		C2		C3	
	50km/h	30 km/h	50km/h	30 km/h	50km/h	30 km/h
**MEAN**	0.00	4.11	10.19	12.71	13.56	16.00
**SD**	0.00	0.18	0.45	0.56	0.60	0.71
**-95% CI**	0.00	3.93	9.74	12.15	12.96	15.29
**+95% CI**	0.00	4.29	10.64	13.28	14.17	16.71
	**C4**		**C5**		**C6**	
	50km/h	30 km/h	50km/h	30 km/h	50km/h	30 km/h
**MEAN**	18.70	20.98	15.28	17.27	19.71	21.08
**SD**	0.83	0.93	0.68	0.77	0.87	0.94
**-95% CI**	17.87	20.05	14.60	16.50	18.83	20.15
**+95% CI**	19.53	21.91	15.96	18.03	20.58	22.02

C1: no cycle path; C2: cycle path separated from traffic by marked white lines; C3: cycle path separated from traffic with a curb, not separated from walking path by color; C4: cycle path separated from traffic with a hedge, not separated from walking path by color; C5: cycle path separated from traffic with a curb, separated from walking path by color; C6: cycle path separated from traffic with a hedge, separated from walking path by color.
