# Supplementary material for: Which environmental factors most strongly influence a street’s appeal for bicycle transport among adults? A conjoint study using manipulated photographs
Source: Int J Health Geogr. 2016 Sep 1;15(1):31. doi: 10.1186/s12942-016-0058-4 (PMC5007833; doi:10.1186/s12942-016-0058-4)
Supplement: Supplementary file 2 — 10.1186/s12942-016-0058-4 Interaction effect between cycle path type and vegetation. [file 12942_2016_58_MOESM2_ESM.pdf]

Additional file 2 - Interaction effect between cycle path type and vegetation

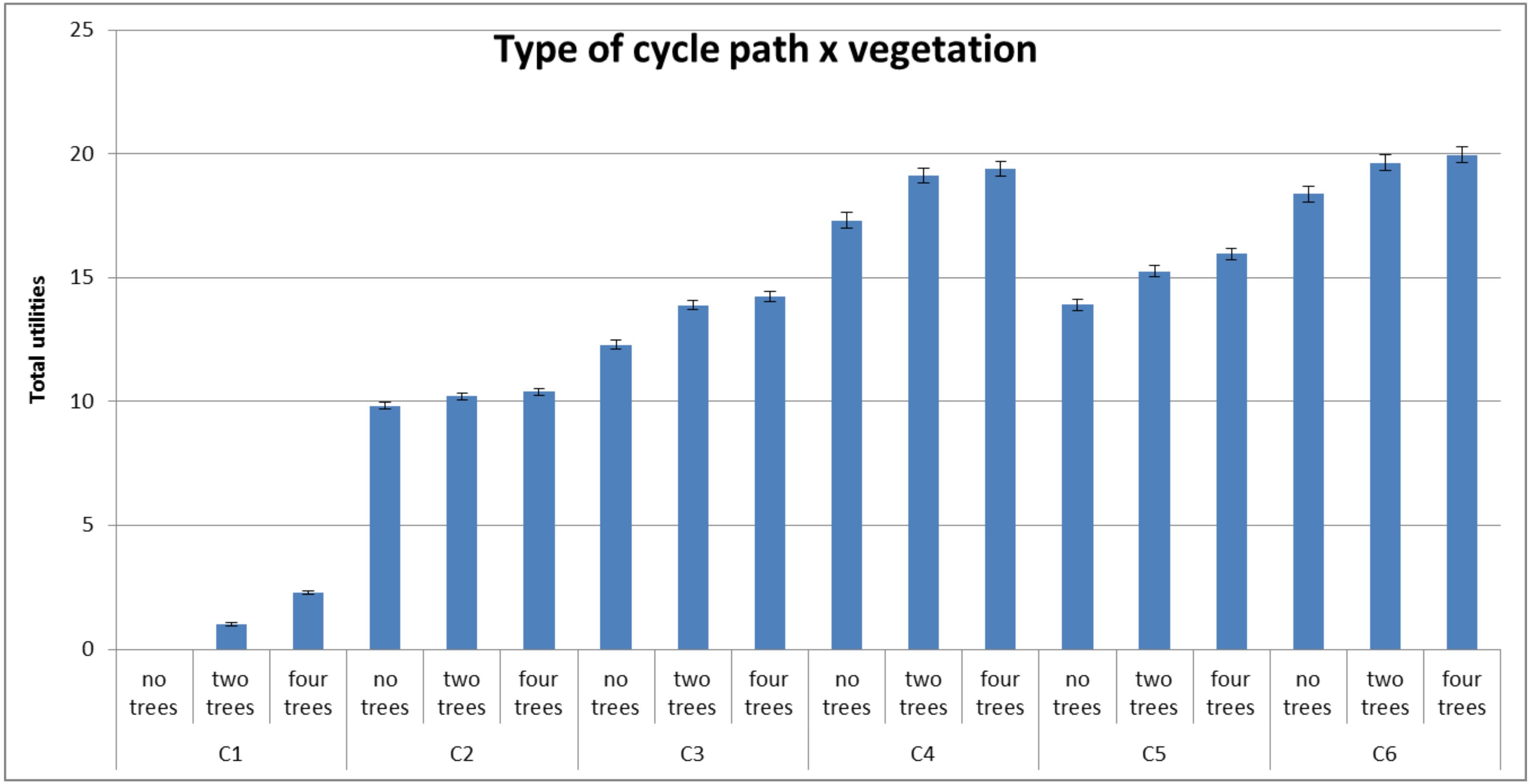

Figure B.1. Interaction effect between type of cycle path and vegetation (chi-square= 27.78; p=0.002)

Table B.1. Interaction effect between type of cycle path and vegetation (chi-square= 27.78; p=0.002)

	C1			C2			C3			C4			C5			C6		
	no trees	two trees	four trees	no trees	two trees	four trees	no trees	two trees	four trees	no trees	two trees	four trees	no trees	two trees	four trees	no trees	two trees	four trees
MEAN	0.00	1.00	2.30	9.83	10.22	10.39	12.30	13.90	14.24	17.30	19.12	19.39	13.91	15.26	15.96	18.37	19.64	19.96
SD	0.00	1.54	1.71	3.09	3.02	2.80	4.32	4.25	4.60	7.08	6.88	6.85	5.03	4.97	5.14	7.45	6.98	7.10
-95% CI	0.00	0.94	2.23	9.69	10.08	10.27	12.11	13.71	14.04	16.99	18.82	19.09	13.69	15.04	A5.73	18.04	19.33	19.64
+95% CI	0.00	1.07	2.38	9.97	10.35	10.52	12.49	14.09	14.44	17.62	19.43	19.69	14.13	15.48	16.19	18.70	19.95	20.27

C1: no cycle path; C2: cycle path separated from traffic by marked white lines; C3: cycle path separated from traffic with a curb, not separated from walking path by color; C4: cycle path separated from traffic with a hedge, not separated from walking path by color; C5: cycle path separated from traffic with a curb, separated from walking path by color; C6: cycle path separated from traffic with a hedge, separated from walking path by color.
