# Supplementary material for: Which environmental factors most strongly influence a street’s appeal for bicycle transport among adults? A conjoint study using manipulated photographs
Source: Int J Health Geogr. 2016 Sep 1;15(1):31. doi: 10.1186/s12942-016-0058-4 (PMC5007833; doi:10.1186/s12942-016-0058-4)
Supplement: Supplementary file 3 — 10.1186/s12942-016-0058-4 Interaction effect between cycle path type and traffic density. [file 12942_2016_58_MOESM3_ESM.pdf]

Additional file 3 - Interaction effect between cycle path type and traffic density

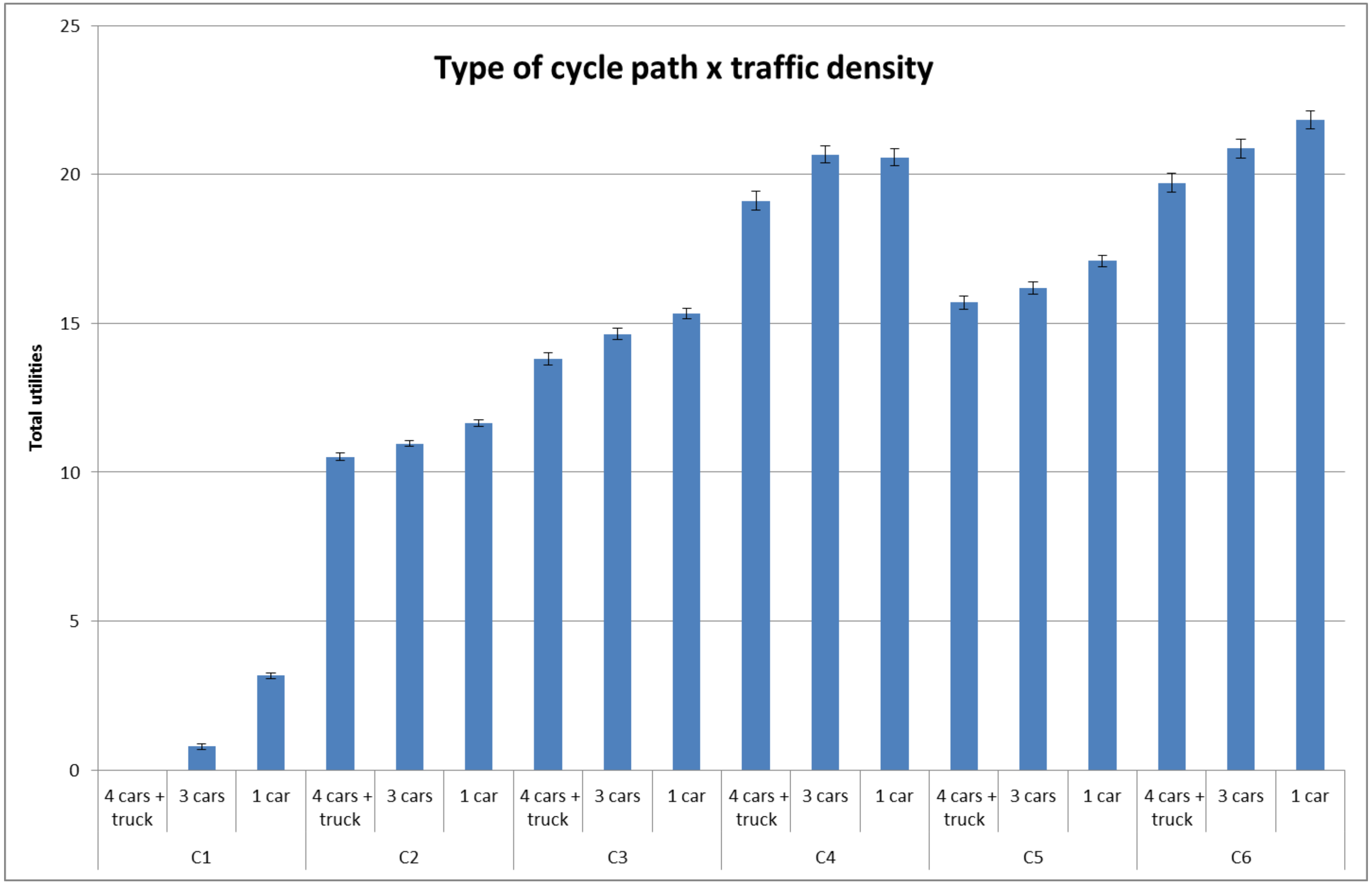

Figure C.1. Interaction effect between type of cycle path and traffic density

Table C.1. Interaction effect between type of cycle path and traffic density

	C1			C2			C3			C4			C5			C6		
	4 cars + truck	3 cars	1 car	4 cars + truck	3 cars	1 car	4 cars + truck	3 cars	1 car	4 cars + truck	3 cars	1 car	4 cars + truck	3 cars	1 car	4 cars + truck	3 cars	1 car
MEAN	0.00	0.79	3.17	10.52	10.96	11.65	13.81	14.64	15.31	19.11	20.67	20.57	15.69	16.17	17.10	19.72	20.86	21.82
SD	0.00	2.18	2.32	2.94	2.28	2.69	4.50	4.26	3.91	7.11	6.52	6.40	5.27	4.67	4.30	7.21	6.88	6.87
-95% CI	0.00	0.70	3.06	10.39	10.86	11.53	13.61	14.45	15.14	18.80	20.38	20.29	15.46	15.96	16.91	19.40	20.56	21.51
+95% CI	0.00	0.89	3.27	10.65	11.06	11.77	14.01	14.83	15.49	19.43	20.96	20.85	15.93	16.38	17.29	20.04	21.17	22.12

C1: no cycle path; C2: cycle path separated from traffic with lines, not separated from walking path; C3: cycle path separated from traffic with a curb, not separated from walking path; C4: cycle path separated from traffic with a hedge, not separated from walking path; C5: cycle path separated from traffic with a curb, separated from walking path by color; C6: cycle path separated from traffic with a hedge, separated from walking path by color.
