# Supplementary material for: Which environmental factors most strongly influence a street’s appeal for bicycle transport among adults? A conjoint study using manipulated photographs
Source: Int J Health Geogr. 2016 Sep 1;15(1):31. doi: 10.1186/s12942-016-0058-4 (PMC5007833; doi:10.1186/s12942-016-0058-4)
Supplement: Supplementary file 4 — 10.1186/s12942-016-0058-4 Interaction effect between cycle path type and evenness. [file 12942_2016_58_MOESM4_ESM.pdf]

Additional file 4 - Interaction effect between cycle path type and evenness

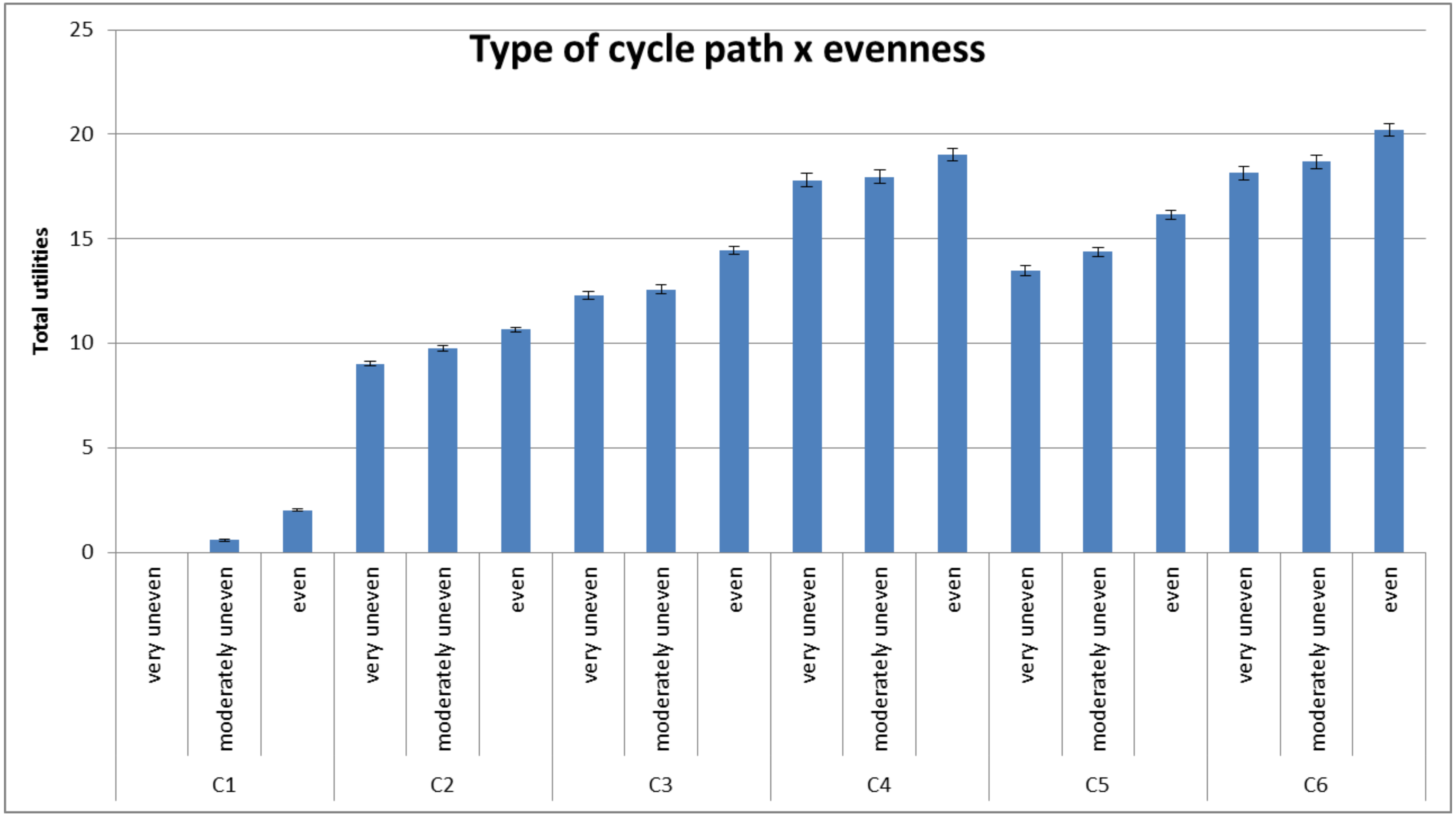

Figure D.1. Interaction effect between type of cycle path and evenness of the cycle path surface.

Table D.1. Interaction effect between type of cycle path and evenness of the cycle path surface.

	C1			C2			C3			C4			C5			C6		
	very uneven	moderately uneven	even	very uneven	moderately uneven	even	very uneven	moderately uneven	even	very uneven	moderately uneven	Even	very uneven	moderately uneven	even	very uneven	moderately uneven	even
MEAN	0.00	0.59	2.02	9.02	9.75	10.65	12.28	12.59	14.43	17.81	17.97	19.05	13.47	14.38	16.15	18.15	18.69	20.19
SD	0.00	1.33	1.56	2.68	3.26	2.98	4.12	4.49	4.19	7.28	6.83	6.63	5.15	4.83	4.70	7.22	7.35	6.69
-95% CI	0.00	0.53	1.95	8.91	9.60	10.52	12.10	12.39	14.25	17.49	17.67	18.75	13.24	14.16	15.94	17.83	18.37	19.90
+95% CI	0.00	0.64	2.09	9.14	9.89	10.78	12.46	12.79	14.62	18.13	18.28	19.34	13.70	14.59	16.36	18.47	19.02	20.49

C1: no cycle path; C2: cycle path separated from traffic by marked white lines; C3: cycle path separated from traffic with a curb, not separated from walking path by color; C4: cycle path separated from traffic with a hedge, not separated from walking path by color; C5: cycle path separated from traffic with a curb, separated from walking path by color; C6: cycle path separated from traffic with a hedge, separated from walking path by color.
