# Supplementary material for: Which environmental factors most strongly influence a street’s appeal for bicycle transport among adults? A conjoint study using manipulated photographs
Source: Int J Health Geogr. 2016 Sep 1;15(1):31. doi: 10.1186/s12942-016-0058-4 (PMC5007833; doi:10.1186/s12942-016-0058-4)
Supplement: Supplementary file 5 — 10.1186/s12942-016-0058-4 Interaction effect between speed bump and traffic density. [file 12942_2016_58_MOESM5_ESM.pdf]

## Additional file 5 - Interaction effect between speed bump and traffic density

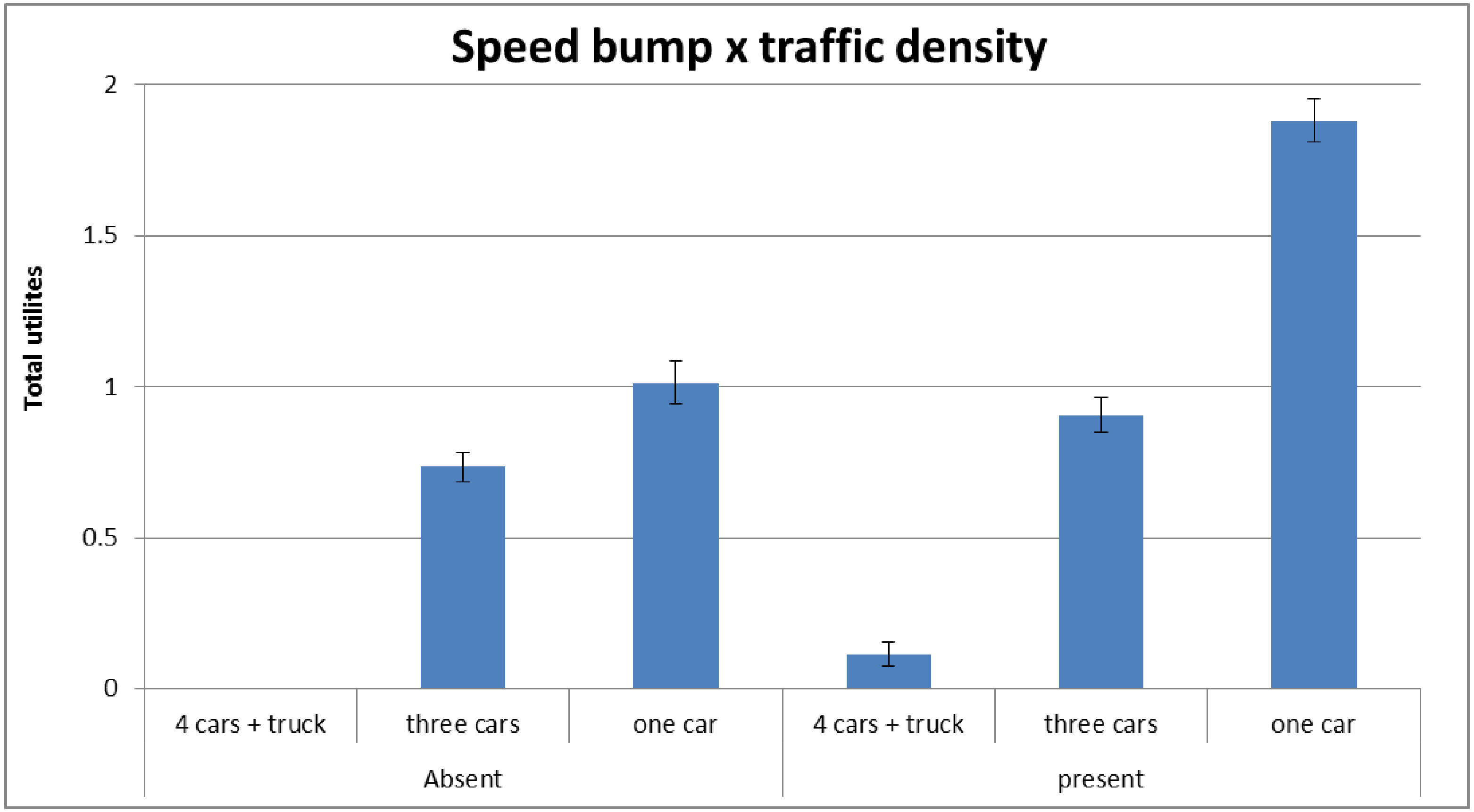


*Figure E.1. Interaction effect between speed bump and traffic density*

*Table E.1. Interaction effect between speed bump and traffic density*

| | Absent | | | Present | | |
|---|---|---|---|---|---|---|
| | 4 cars + truck | three cars | one car | 4 cars + truck | three cars | one car |
| MEAN | 0.00 | 0.73 | 1.01 | 0.11 | 0.91 | 1.88 |
| SD | 0.00 | 1.13 | 1.65 | 0.89 | 1.29 | 1.61 |
| -95% CI | 0.00 | 0.68 | 0.94 | 0.08 | 0.85 | 1.81 |
| +95% CI | 0.00 | 0.78 | 1.09 | 0.15 | 0.96 | 1.95 |
