# Supplementary material for: Which environmental factors most strongly influence a street’s appeal for bicycle transport among adults? A conjoint study using manipulated photographs
Source: Int J Health Geogr. 2016 Sep 1;15(1):31. doi: 10.1186/s12942-016-0058-4 (PMC5007833; doi:10.1186/s12942-016-0058-4)
Supplement: Supplementary file 6 — 10.1186/s12942-016-0058-4 Interaction effect between vegetation and general upkeep. [file 12942_2016_58_MOESM6_ESM.pdf]

Additional file 6 - Interaction effect between vegetation and general upkeep

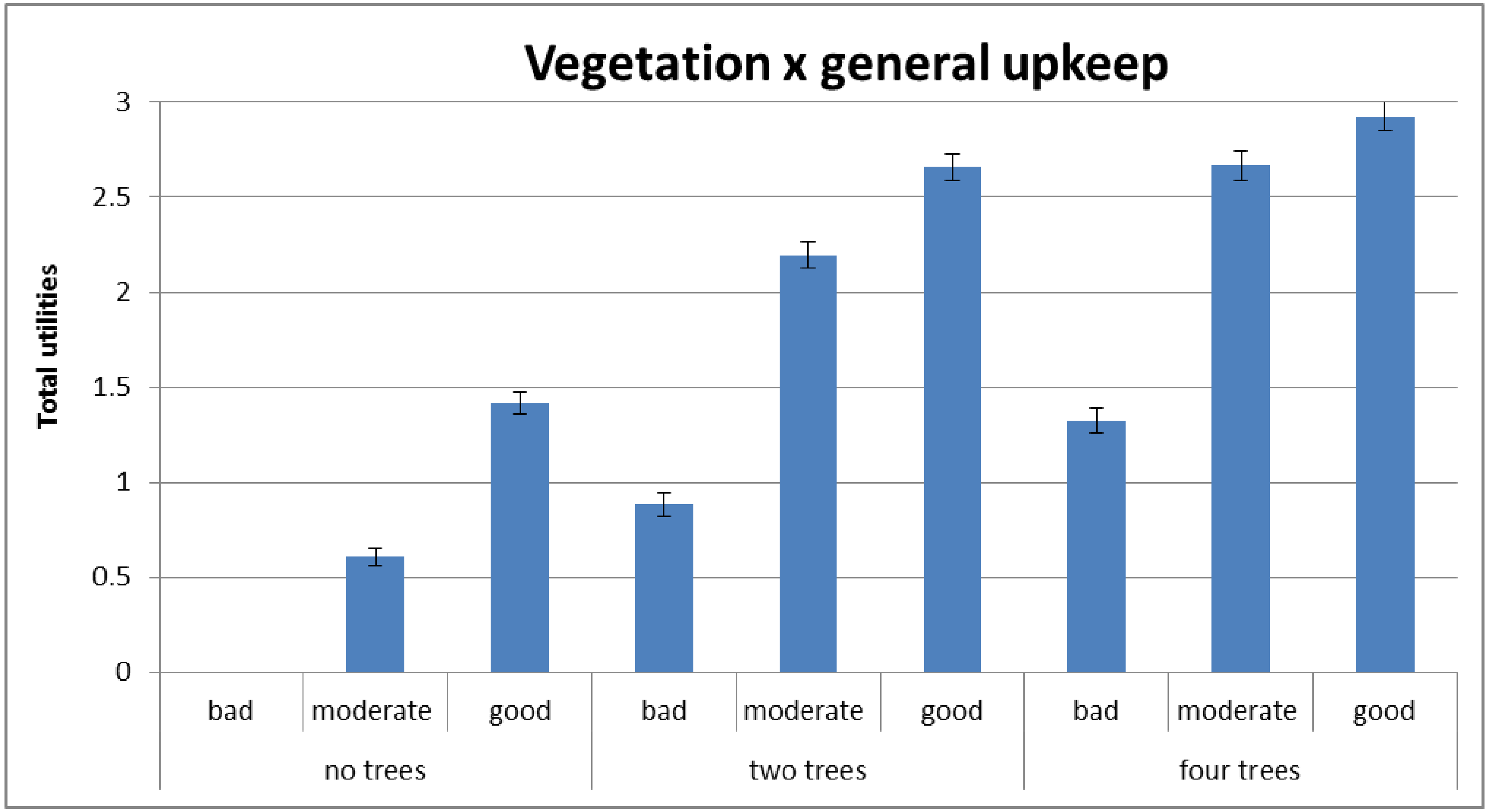

Figure F.1. Interaction effect between vegetation and general upkeep

Table F.1. Interaction effect between vegetation and general upkeep

	no trees			two trees			four trees		
	bad	moderate	good	bad	moderate	good	bad	moderate	Good
MEAN	0.00	0.60	1.42	0.88	2.20	2.66	1.32	2.67	2.93
SD	0.00	1.09	1.32	1.32	1.50	1.54	1.45	1.74	1.72
-95% CI	0.00	0.56	1.36	0.82	2.13	2.59	1.26	2.59	2.85
+95% CI	0.00	0.00	1.48	-0.29	-2.67	-3.54	-1.02	2.75	-3.87
